# Oral vitamin D supplementation induces transcriptomic changes in rectal mucosa that are linked to anti-tumour effects

**DOI:** 10.1186/s12916-021-02044-y

**Published:** 2021-08-03

**Authors:** P. G. Vaughan-Shaw, G. Grimes, J. P. Blackmur, M. Timofeeva, M. Walker, L. Y. Ooi, Victoria Svinti, Kevin Donnelly, F. V. N. Din, S. M. Farrington, M. G. Dunlop

**Affiliations:** 1 MRC Human Genetics Unit, Institute of Genetics and Cancer, University of Edinburgh, Crewe Road, Edinburgh, EH4 2XU UK; 2 Cancer Research UK Edinburgh Centre, Institute of Genetics and Cancer, University of Edinburgh, Edinburgh, UK; 3 DIAS, Danish Institute for Advanced Study, Department of Public Health, University of Southern Denmark, Odense, Denmark; 4 Deanery of Molecular, Genetic & Population Health Sciences, in the College of Medicine & Veterinary Medicine, University of Edinburgh, Edinburgh, UK; 5 Department of Pathology, National University Hospital, National University Health System, Singapore, Singapore

**Keywords:** Vitamin D, Colorectal cancer, Gene expression, Biomarker

## Abstract

**Background:**

The risk for several common cancers is influenced by the transcriptomic landscape of the respective tissue-of-origin. Vitamin D influences in vitro gene expression and cancer cell growth. We sought to determine whether oral vitamin D induces beneficial gene expression effects in human rectal epithelium and identify biomarkers of response.

**Methods:**

Blood and rectal mucosa was sampled from 191 human subjects and mucosa gene expression (HT12) correlated with plasma vitamin D (25-OHD) to identify differentially expressed genes. Fifty subjects were then administered 3200IU/day oral vitamin D3 and matched blood/mucosa resampled after 12 weeks. Transcriptomic changes (HT12/RNAseq) after supplementation were tested against the prioritised genes for gene-set and GO-process enrichment. To identify blood biomarkers of mucosal response, we derived receiver-operator curves and C-statistic (AUC) and tested biomarker reproducibility in an independent Supplementation Trial (BEST-D).

**Results:**

Six hundred twenty-nine genes were associated with 25-OHD level (*P* < 0.01), highlighting 453 GO-term processes (FDR<0.05). In the whole intervention cohort, vitamin D supplementation enriched the prioritised mucosal gene-set (upregulated gene-set *P* < 1.0E−07; downregulated gene-set *P* < 2.6E−05) and corresponding GO terms (*P* = 2.90E−02), highlighting gene expression patterns consistent with anti-tumour effects. However, only 9 individual participants (18%) showed a significant response (NM gene-set enrichment *P* < 0.001) to supplementation. Expression changes in *HIPK2* and *PPP1CC* expression served as blood biomarkers of mucosal transcriptomic response (AUC=0.84 [95%CI 0.66–1.00]) and replicated in BEST-D trial subjects (*HIPK2* AUC=0.83 [95%CI 0.77–0.89]; *PPP1CC* AUC=0.91 [95%CI 0.86–0.95]).

**Conclusions:**

Higher plasma 25-OHD correlates with rectal mucosa gene expression patterns consistent with anti-tumour effects, and this beneficial signature is induced by short-term vitamin D supplementation. Heterogenous gene expression responses to vitamin D may limit the ability of randomised trials to identify beneficial effects of supplementation on CRC risk. However, in the current study blood expression changes in *HIPK2* and *PPP1CC* identify those participants with significant anti-tumour transcriptomic responses to supplementation in the rectum. These data provide compelling rationale for a trial of vitamin D and CRC prevention using easily assayed blood gene expression signatures as intermediate biomarkers of response.

**Supplementary Information:**

The online version contains supplementary material available at 10.1186/s12916-021-02044-y.

## Background

Vitamin D deficiency is associated with the risk of several common cancers, the strongest evidence supporting a link between vitamin D and colorectal cancer [1, 2]. However, a causal association has yet to be convincingly demonstrated, because the available observational evidence may be participant to several potential confounders. Environmental risk factors associated with CRC are also associated with vitamin D status (i.e. co-causality; e.g. physical activity), while CRC or its treatment may itself lower plasma vitamin D levels (i.e. reverse causation). However, a recent randomised-control trial (RCT) reported an association between supplementation, vitamin D receptor genotype, and risk of colorectal adenoma, supporting the premise that the beneficial effect may be causal [3]. Meanwhile, vitamin D-related genetic variation has been shown to influence the association between 25-OHD level and CRC survival [4–6], with a recent meta-analysis of RCT data strongly supporting a causal effect for vitamin D supplementation on CRC mortality [7, 8].

Differences in gene expression have been reported in CRC and adenoma tissue relative to normal colorectal tissue [9–12], with genes involved in metabolism, transcription, and translation and cellular processes commonly altered [13]. Recent transcriptome wide association studies confirm the importance of gene expression in carcinogenesis [14, 15]. Vitamin D broadly influences gene expression through activation of the ligand-activated transcription factor *VDR*, which has been shown to influence cancer cell growth in vitro [16]. Therefore, investigation of gene expression in the colorectum in the context of vitamin D status or supplementation may provide fresh insight into mechanisms underlying the relationship between CRC and vitamin D. Recent evidence suggests one mechanism may be that 1,25-dihydroxyvitamin D3 modulates immune and inflammatory pathway genes in large bowel epithelium [17]. However, differential expression in response to high dose 1,25-dihydroxyvitamin D3 may not accurately reflect the relationship between vitamin D status and gene expression at normal or low vitamin D levels, or in response to regular vitamin D3, the most commonly used vitamin D supplement.

We investigated whether circulating vitamin D concentration is associated with differential gene expression in rectal normal mucosa using a 2-Phase approach with validation of putative biomarkers in an independent study dataset. We directly assayed total 25-OHD, which reflects both dietary intake and skin synthesis of vitamin D [18, 19] and investigated its relationship with gene expression in normal mucosa, assessed by microarray. In the Phase 1 correlative study we sought to identify a prioritised list of differentially expressed genes associated with 25-OHD level. In Phase 2, we conducted a study in human volunteers who were supplemented with oral vitamin D to determine whether the corresponding transcriptomic response was induced in vivo. Using blood peripheral blood mononuclear cells (PBMC) transcriptomic analysis, we also identified potential blood biomarkers that indirectly indicate a beneficial response in the host rectal mucosa.

## Methods

### Study population

Participants recruited to Phase 1 of the Scottish Vitamin D study (see Additional file 1: Protocol) (*n* = 191) underwent sampling of the blood and normal rectal mucosa by rigid sigmoidoscopic biopsy. Eligibility criteria included age over 16, ability to perform informed consent, absence of bleeding risk (e.g. coagulopathy, anti-coagulants) and absence of acute colorectal/ano-rectal pathology (e.g. peritonitis, diverticulitis, recent colorectal surgery, anal fissure). RNA was extracted for gene expression analysis from matched contemporaneous NM and peripheral blood mononuclear cells (PBMCs). Plasma was collected at the same time for vitamin D analysis, and DNA was extracted from whole blood for genotyping.

**Fig. 1:**
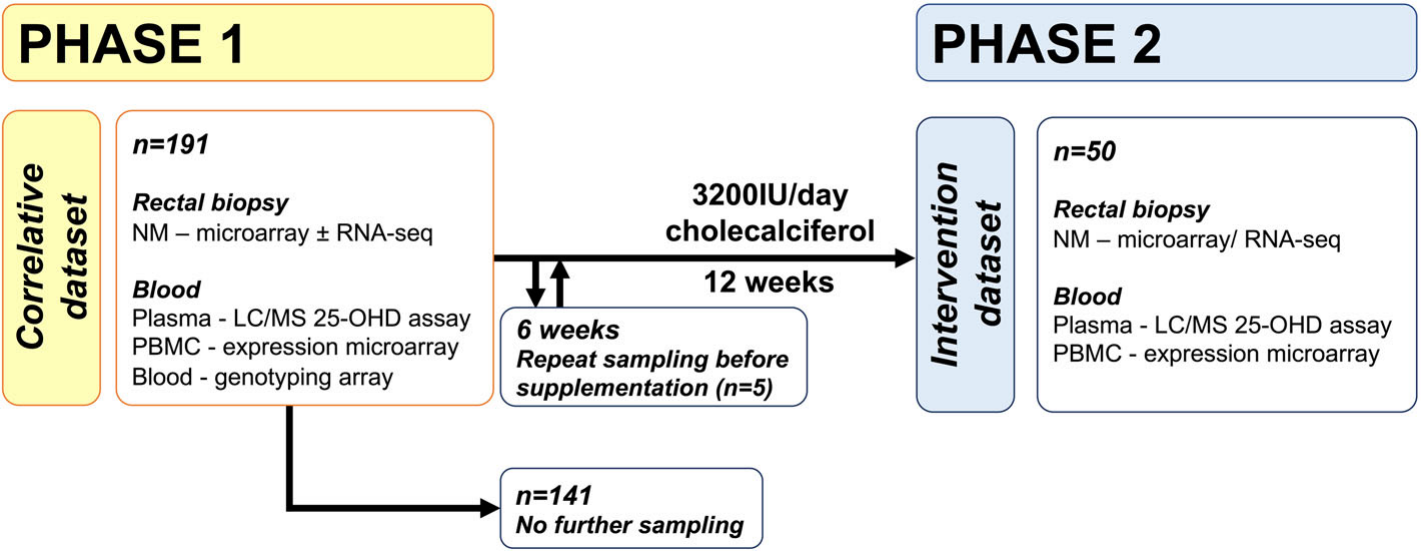
Summary of SCOVIDS study protocol. An unselected subset of Phase 1 participants (i.e. selection not based on 25-OHD or baseline gene expression) proceeded to Phase 2 and were given 3200IU cholecalciferol per day. Of these, 5 participants were sampled 6 weeks after initial sampling and without supplementation to provide a control dataset, after which they proceeded to 12 week’s supplementation and final sampling

All eligible participants from Phase 1 were invited to proceed to Phase 2 which was an intervention study. Five of the 50 recruited participants to Phase 2 underwent interval sampling *before* starting supplementation to assess for longitudinal changes in expression before treatment. All Phase 2 participants were then administered vitamin D supplementation and underwent repeat NM, PBMC, and 25-OHD sampling after 12 weeks’ 3200IU/day cholecalciferol (Fultium-D3) supplementation (Fig. 1). No vitamin D supplementation outside of the study protocol was allowed, and concordance with the treatment protocol was assessed through a dose diary and pharmacy log of unused tablets (compliance of 98% of total doses taken achieved). Demographic and clinical data were prospectively collected from patient case notes.

### Sample size considerations

There were no available published data on which to base investigation of the sample size required to determine an association between vitamin D status and global gene expression in normal mucosa. Thus, a formal sample size estimation was not possible.

### Blood and mucosa sampling

Participants were sampled in outpatient clinic or during minor surgical procedures. No participant received cleansing oral mechanical bowel preparation. Blood was sampled by standard venepuncture of a peripheral arm vein, with plasma and PBMCs extracted (Additional file 2: Supplementary Methods [20–29]). A separate blood sample was taken to allow extraction of blood leukocyte DNA and genotyping of functionally relevant vitamin D receptor (*VDR*) polymorphisms (rs1544410, rs10735810, rs7975232, rs11568820 [26–28]) and vitamin D pathway SNPs associated with 25-OHD level (rs2282679, rs10741657, rs2228570, rs6013897 [25]). Normal rectal mucosa (NM) was sampled at the same time via rigid sigmoidoscopic rectal biopsy. NM and PBMC samples were immediately placed in RNAlater and kept immersed for 24–72 h prior to RNA extraction or storage at −80°C.

### Plasma vitamin D assay

All plasma samples were measured to a standard, validated, and published protocol by a single laboratory [30]. Total 25-OHD was measured by liquid chromatography tandem mass spectrometry.

### Assessment of gene expression

RNA was extracted and purified from NM and blood PBMC using a proprietary RNA extraction kit. Gene expression profiling was undertaken using the Illumina HumanHT-12v4.0 Expression BeadChip Arrays and IScan NO660 scanner, providing coverage of 47,231 transcripts and >31,000 annotated genes. For RNA sequencing, whole-genome transcriptomic patterns were analysed on total RNA from selected normal mucosa samples extracted as described above. RNA was sequenced on the Illumina HiSeq 2500 platform in “rapid mode” with 150bp paired-end reads in a single batch. Transcript indexing and quantification from RNA-seq reads was performed using Salmon v1.1.0 [21].

### Statistical analysis

All statistical analysis was undertaken in R [29]. In Phase 1, linear regression modelling was used to test association between 25-OHD level and NM gene expression, adjusting for age, gender, CRC status and anaesthetic status (i.e. sampled under general anaesthetic). *VDR* haplotype was included in a secondary model for a subset of participants. Genes associated with 25-OHD level at significance level *P* < 0.01 were termed the ‘candidate gene-set’ and taken forward for testing in the intervention dataset (Phase 2). In Phase 2, differences in 25-OHD level before and after vitamin D supplementation were investigated using paired Wilcoxon rank-sum test and differential gene expression analysis in response to vitamin D supplementation performed using the *lmFit* and *eBayes* functions within the ‘limma’ package [31] producing the intervention (Phase 2) dataset. Ranked lists of differentially expressed genes were assessed for functional relevance using the ‘GOrilla', Gene Ontology enRIchment anaLysis and visuaLizAtion tool [32] and replicated using the gseGO function within the ‘clusterProfiler’ package in R [33]. Process ontologies were investigated using gene lists ranked by coefficient (Phase 1) or fold-change (Phase 2).

We tested the Phase 2 dataset (i.e. response to supplementation) for enrichment of the Phase 1 candidate gene-set and top-ranked GO terms. Directional gene-set testing was performed in R, using the gene-setTest function in the ‘limma’ package [34]. We performed technical replication by performing gene-set enrichment testing on differential expression data derived from RNA-seq analysis of the same NM samples from the intervention cohort.

To identify biomarkers of response, we performed participant-level gene-set enrichment testing with a ‘response’ to supplementation defined as enrichment (*P* < 0.001 given *n* = 50 subjects) of the candidate gene-set after supplementation. Then, differentially expressed genes in the *blood* between those with/without rectal NM response were tested for enrichment of the candidate gene-set. Logistic regression testing sought to identify potential blood biomarkers of response and utility of blood biomarkers was calculated using receiver operator curves and C statistic. Finally, we sought to validate putative biomarkers of response in an independent blood gene expression dataset derived from the ‘Biochemical Efficacy and Safety Trial of Vitamin D’ (BEST-D) study [35] (https://www.ebi.ac.uk/arrayexpress/files/E-MTAB-6246/).

## Results

### Mucosal gene expression signature associated with higher 25-OHD level consistent with anti-tumour effects

**Fig. 2:**
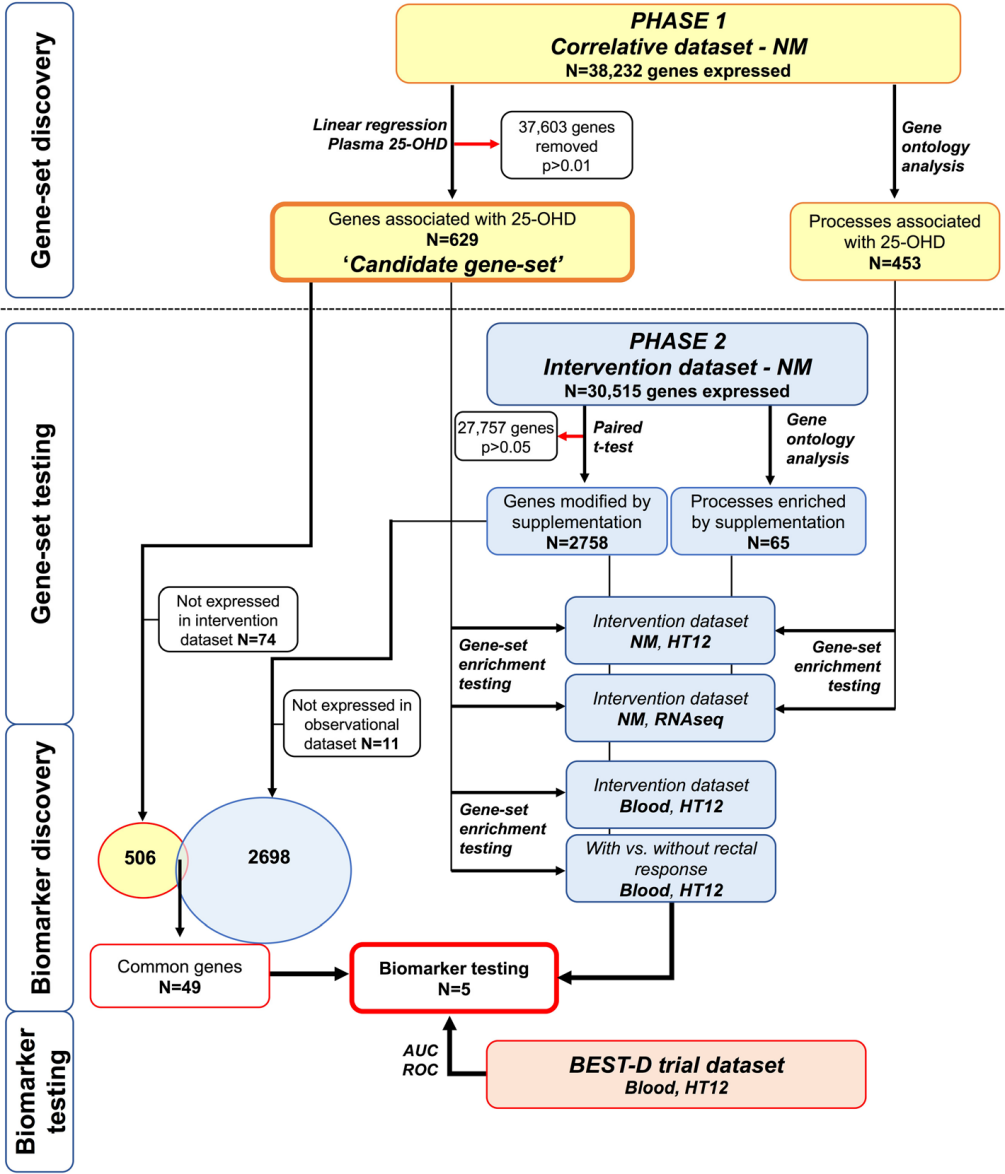
Analysis flowchart and gene-set selection for biomarker assessment

In Phase 1, 191 participants underwent rectal mucosal biopsy and blood sampling (Table 1). 25-OHD was nominally associated with expression of 629 probes (*P* < 0.01), termed the ‘candidate gene-set’ (‘Gene-set discovery’ Fig. 2).

**Table 1:** Baseline characteristics, sampling variables and vitamin D status in included participants

	PHASE 1 Correlative dataset	PHASE 2 Intervention dataset
N	191	50
Age (median, range)	63 (24-89) years	66 (24-88) years
Gender (male)	101 (53%)	26 (52%)
Diagnosis		
Pre-operative; colorectal cancer*	57	1
None (healthy)	63	20
Past medical history of CRC*	23	21
Minor anorectal pathology, no CRC^†^	45	7
Abdominal tumour (not colorectal)	3	1
Sampled under general anaesthetic	89	3
Median 25-OHD –Baseline	40 (IQR 37) nmol/l	36 (IQR 31) nmol/l
Median 25-OHD –After supplementation	NA	89 (IQR 33.5) nmol/l

Ethnic background for Phase 1 participants were as follows: 183 European, 2 American, 3 African, 3 Asian. * Pre-operative patients had not undergone any neo-adjuvant chemo/radiotherapy at time of sampling. Patients with past history of CRC were previously treated with curative resection +/- adjuvant chemotherapy and no evidence of recurrence at time of recruitment. No significant differences in gene expression were identified according to participant diagnosis. † Full diagnosis list given in Additional file 3: Table S1

No individual probe was significantly associated with 25-OHD after adjustment for genome-wide multiple testing (Additional file 3: Table S2), yet the top 3 hits have previous reported association with colorectal tumourigenesis *CNN1* [36], *COX7A1* [37] and *PIP5K1C* [38]. Gene ontology analysis demonstrated significant enrichment of 453 processes (Additional file 3: Table S3) with many highly relevant to carcinogenesis e.g. ‘regulation of cell migration’ (FDR=7.55E-08), ‘regulation of programmed cell death’ (FDR=5.38E-03) and ‘regulation of cell differentiation’ (FDR=2.55E-05). Several genes from the candidate gene-set with higher expression associated with higher 25-OHD are included in enriched GO ontology terms relevant to carcinogenesis and have reported tumour suppressor activity (e.g. *FOXOs*, *CAV1*, *LRP1*, Additional file 3: Tables S4, S5 [39–58]). This suggests that the NM gene expression signature, i.e. Phase 1 ‘candidate gene-set’, associated with higher 25-OHD level is consistent with anti-tumour effects.

### Oral vitamin D supplementation enriches anti-tumour expression signature in normal rectal mucosa

In Phase 2, 50 participants were administered vitamin D supplementation and underwent repeat sampling after 12-weeks. Post hoc analysis revealed age, gender and baseline 25-OHD to be similar between Phase 1/2 participants (*P* > 0.05). Supplementation induced an increase in plasma 25-OHD after 12 weeks (median plasma 25-OHD before/after supplementation was 36nmol/l, 89nmol/l; *P* = 2.5E−09), with a suggestive association between genotype at two vitamin D pathway SNPs (rs2282679, rs2228570) and 25-OHD fold-change after supplementation (Additional file 3: Table S6).

No individual gene from the candidate gene-set showed significant differential expression after adjustment for multiple testing (Additional file 3: Table S7). However, testing of the Phase 1 candidate gene-set showed significant enrichment after supplementation (upregulated gene-set *P* < 1.0E−07; downregulated gene-set 2.8E−05, see ‘Gene-set testing’ Fig. 2, Table 2, Additional file 3: Figure S1), confirmed in RNA-seq data. Gene-set testing of a candidate gene-set from Phase 1 adjusted for VDR haplotypes, also showed significant enrichment (*P* < 0.0001).

**Table 2:** Gene-set testing for enrichment of the candidate gene-set from Phase 1 after supplementation in Phase 2 and the BEST-D study

PHASE 1 Correlative dataset	SCOVIDS Phase 2				BEST-D trial
	Intervention dataset, NM All participants		Intervention dataset, blood, HT12		Blood, HT12
	HT12	RNA-seq	All participants	With vs. without rectal response	All participants
Candidate gene-set: positive association with 25-OHD level	*P* < 1.0E−07 *n* = 349	2.05E−07 *n* = 242	*P* = 3.89E−13 *n* = 239	*P* = 3.65E−12 *n* = 291	*P* = 4.72E−06 *n* = 191
Candidate gene-set: negative association with 25-OHD level	*P* = 2.8E−05 *n* = 206	6.87E−09 *n* = 155	*P* = 9.60E−05 *n* = 185	*P* = 3.50E−17 *n* = 217	*P* = 0.02 *n* = 130

*NM* normal mucosa. *P* value given for directional gene-set enrichment test of whether Phase 1 candidate gene-set showed greater change after supplementation when compared to randomly chosen genes. n = number of genes tested. Rectal response is defined by candidate gene-set enrichment in NM *P* < 0.001. To explore the role of VDR genotype in modifying the association between 25-OHD and gene expression, VDR haplotypes were derived for the four genotyped VDR polymorphisms, rs1544410, rs10735810, rs7975232, rs11568820, using BEAGLE software (version 3.3.2) with standard settings [59] and used as additional covariates in the linear regression model in a subset of 125 participants, resulting in a smaller candidate gene-set which remained significantly enriched after supplementation (*P* < 0.0001)

Enrichment of the candidate gene-set was not associated with 25-OHD response to supplementation, with those with the lowest 25-OHD FCs still showing gene-set enrichment (Additional file 3: Figure. S1). Meanwhile, there was no enrichment of the candidate gene-set in interval NM samples taken *before* commencement of supplementation indicating it is a treatment effect (median interval 8 weeks, Additional file 3: Figure S1).

Of the 629 candidate genes associated with circulating vitamin D in the Phase 1 gene-set, 55 had nominally significant expression change after supplementation (*P* < 0.05). Concordance in direction of effect between the coefficient of association with plasma 25-OHD level and expression change after supplementation was observed in 49 of these (R0.93, *P* < 2.2E**−**16, Additional file 3: Table S7), with these genes taken forward for biomarker discovery (see ‘Biomarker discovery’ section Fig. 2).

### GO term enrichment indicates modulation of anti-tumour effects in normal mucosa by supplementation

Functional annotation of the intervention dataset gene list identified 65 significantly enriched pathways after supplementation, with many terms relevant to carcinogenesis including ‘regulation of programmed cell death (FDR=9.66E-03) and ‘regulation of cell migration’ (FDR=7.83E−03) (Additional file 3: Table S8). Taken together, genes in the top 50 GO terms from Phase 1 were significantly enriched after supplementation (*P* = 2.90E−02). Common processes across both the Phase 1 and Phase 2 datasets included terms relevant to carcinogenesis including ‘regulation of programmed cell death’, ‘regulation of cell migration’ (Additional file 3: Figure S2), demonstrating that biologically relevant patterns of gene expression changes associated with higher 25-OHD level and consistent with anti-tumour effects could be imparted by oral supplementation. Results from GOrilla analysis were replicated using both DAVID and GSEA in R, which confirmed enrichment of common processes depicted in Additional file 3: Figure S2, and direction of effect, with terms consistent with ‘anti-tumour’ effects activated in relation to both higher 25-OHD and after vitamin D supplementation.

### Blood expression biomarkers identify participants with gene expression response to supplementation

We identified 9 individual participants (18%) with a significant response (i.e. candidate gene-set enrichment in NM *P* < 0.001) to supplementation. Of the top 50 ranked GO terms from Phase 1, 43 were enriched in these 9 participants after supplementation indicating a biologically relevant NM gene expression response to supplementation, which was absent in the remaining participants (Additional file 3: Table S9). NM gene expression response was associated with an increased allele risk score of the four functionally relevant *VDR* SNPs (*p* = 0.006), but not with *VDR* gene expression, increased 25-OHD fold-change or baseline 25-OHD when adjusting for multiple testing (Additional file 3: Table S10).

Changes in PBMC gene expression after supplementation reflected those in the rectum, with the Phase 1 candidate gene-set significantly enriched in the blood after supplementation (Table 2). Moderate correlation between blood and rectum fold-change in the 49 genes taken forward for biomarker discovery was seen (R=0.64, *P* = 5.63E−06). When we compared PBMC gene expression after supplementation between those participants *with* and *without* a rectal mucosal gene expression response, the differentially expressed genes in PBMCs were enriched for the candidate gene-set, indicating potential blood biomarkers of mucosal response (Table 2, see ‘Biomarker discovery’ section Fig. 2). Five genes identified from the Phase 1 which were both differentially expressed in NM after supplementation and also differentially expressed in blood between participants *with* and *without* a rectal response to supplementation (*SMEK2*, *HIPK2*, *PPP1C*, *DDR1 and SNX21*), indicating biomarker potential (Table 3). When the genes were combined, a blood expression signature based on the best derived cut-off showed strong utility in predicting NM response (AUC=0.99, 95%CI 0.97–1.00, Additional file 3: Table S11, Figure S3).

**Table 3:** Genes prioritised from Phase 1 correlative dataset modified by supplementation in NM with evidence of potential biomarker utility in PHASE 2 and BEST-D trial

Gene	Phase 1 NM		Phase 2 NM		Phase 2 Blood		Phase 2 With vs. without response, Blood		BEST D trial Blood		BEST-D trial With vs. without response, Blood	
	Coeff.	***P*** value	FC	***P*** value	FC	***P*** value	FC	***P*** value	FC	***P*** value	FC	***P*** value
***SMEK2***	−0.002	0.007	0.93	0.020	0.97	0.272	0.74	0.007	-	-	-	-
***HIPK2***	0.004	0.006	1.17	0.016	1.11	0.068	1.46	0.014	1.07	0.002	1.36	8.47E−12
***PPP1CC***	−0.002	0.007	0.93	0.020	0.91	0.006	0.82	0.047	0.96	0.14	0.61	1.98E−17
***SNX21***	0.004	0.003	1.07	0.047	0.99	0.750	1.26	0.039	1.01	0.27	1.04	0.02
***DDR1***	0.007	0.008	1.14	0.0008	1.03	0.572	0.72	0.040	1.02	0.03	1.04	0.08

*NM* normal mucosa, *FC* fold-change. Results from Phase 1 and Phase 2 analysis given. For Phase 1, coefficient given for association with 25-OHD level. For Phase 2, fold-change and *P* value given for gene expression response to supplementation in NM and in the blood. In final column, blood expression fold-change difference between participants with and without NM response to the candidate gene-set is given

**Fig. 3:**
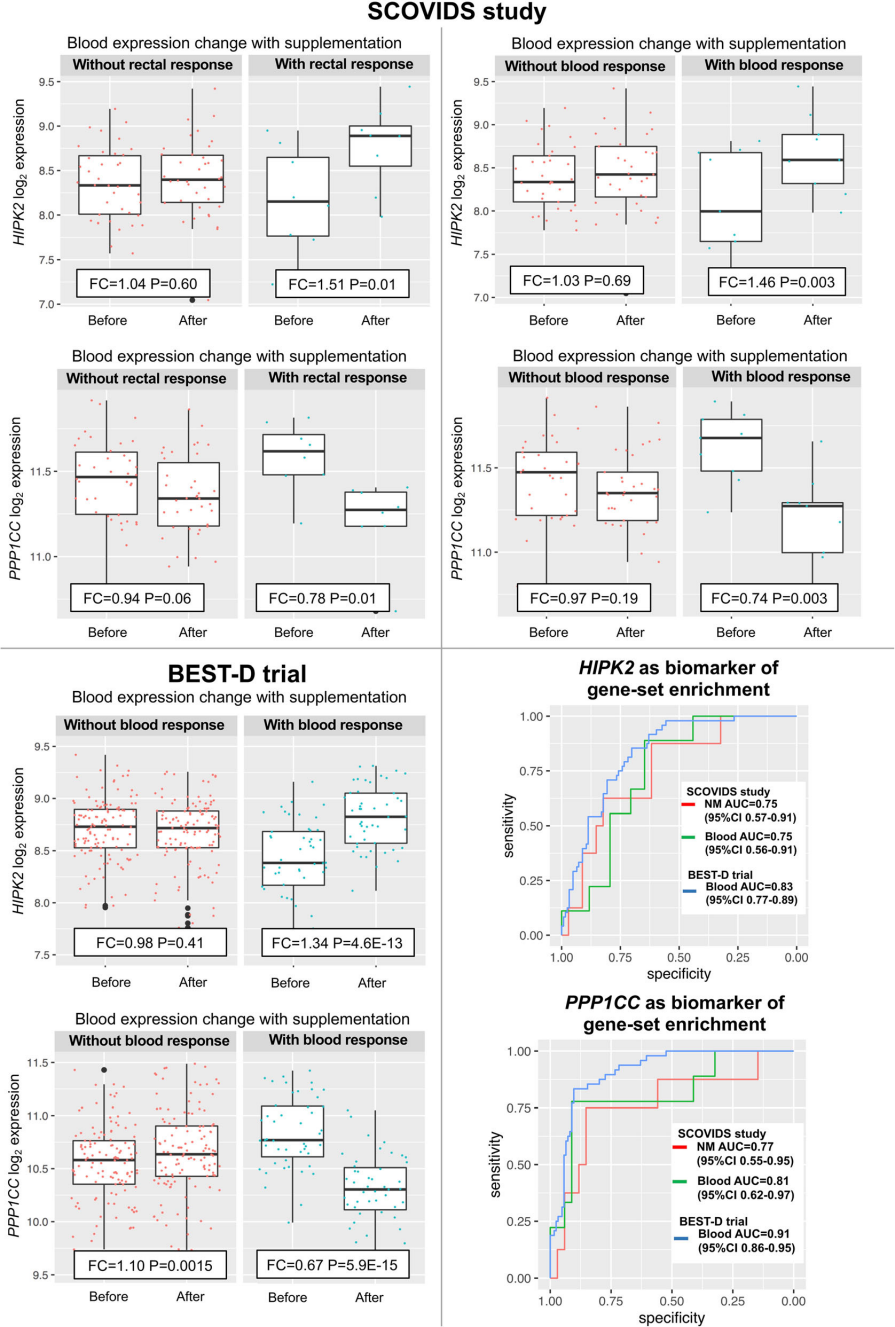
Blood *HIPK2* and *PPP1CC* expression before and after supplementation in SCOVIDS and BEST-D trial with ROC of biomarker utility. Response defined as participant level gene-set enrichment to Phase 1 candidate gene-set from our SCOVIDS study after HIPK2, PPP1CC, SMEK2, DDR1 and SNX21 excluded

We then explored the value of these same genes as blood biomarkers of the *blood* response to supplementation. The *HIPK2* and *PPP1CC* genes (Table 3, Fig. 3) demonstrated the best predictive utility in identifying participants with response in both rectum (AUC=0.84, 95%CI 0.66–1.00) and blood (AUC=0.87, 95%CI 0.71–1.00, Additional file 3: Table S11).

### *HIPK2* and *PPP1CC* are independent biomarkers of expression response to supplementation in the BEST-D trial expression dataset

In the BEST-D trial, 48/172 (28%) participants showed significant enrichment of our Phase 1 candidate gene-set. *HIPK2* expression increased with supplementation in the BEST-D trial (FC=1.07, *p* = 0.002), with the increase greatest in those with a blood response to supplementation as defined by our candidate gene-set (FC=1.34; *P* = 4.6E−13, Fig. 3) and a 1.36 fold-difference in expression change after supplementation between those with/without a response to our candidate gene-set (*P* = 8.48E−12, Table 3). In the current intervention study (SCOVIDS), we observed an average *HIPK2* FC=1.11 in the blood, with 21 (47%) participants showing a FC>1.19, the optimum threshold determined by AUC calculations. In the BEST-D study, 63 (37%) participants taking oral vitamin D supplementation showed a FC>1.19 response, with this signature more prevalent in those taking 4000IU per day (42%), suggesting a dose-response. *HIPK2* expression change showed utility in identifying those with a response to our candidate gene-set, AUC=0.83 (95%CI 0.77–0.89, Fig. 3) and when the threshold from our study was used, *HIPK2* FC>1.19 AUC=0.74 (95%CI 0.66–0.81, Additional file 3: Table S11).

PPP1CC expression showed non-significant decrease after supplementation in the BEST-D cohort overall, but when the participants were stratified by blood response, *PPP1CC* was seen to increase in those without blood response (*P* = 0.0015, Fig. 3) and markedly decreased in those with a blood response (FC=0.67; *P* = 5.9E−15). There was a 0.61 fold-difference in *PPP1CC* expression change between those with and without a response to our candidate gene-set (*P* = 1.98E−17, Table 3). A total of 47 (27%) participants had *PPP1CC* FC<0.76 after supplementation, closely reflecting the 11 (26%) participants in the current intervention study. Crucially, *PPP1CC* expression change after supplementation showed utility as a biomarker of blood response to our candidate gene-set, AUC=0.91 (95%CI=0.86−0.95, Fig. 3) and when using the threshold from the SCOVIDs study, *PPP1CC* FC<0.76 AUC=0.83 (95%CI 0.76−0.89, Additional file 3: Table S11).

## Discussion

This study reveals demonstrable differences in gene expression patterns in normal rectal mucosa correlated with plasma 25-OHD level. These differences are consistent with beneficial effects on processes relevant to colorectal carcinogenesis. Furthermore, we show that oral supplementation with vitamin D induces changes in the prioritised gene list. This indicates that the beneficial expression “signature” is not static, but rather can be modified by oral vitamin D supplementation, at least within the timescale tested here. Although we were not able to directly test cancer endpoints, there is considerable published evidence supporting the premise that enrichment of this favourable gene-set imparts anti-tumour effects.

Homeodomain-interacting protein kinase 2 (*HIPK2*) is a known tumour suppressor gene [60] and the Protein Phosphatase 1 Catalytic Subunit Gamma gene (*PPP1CC*), a published molecular marker of CRC [61]. We found expression changes in these genes in blood to have predictive value in reflecting rectal mucosa response to supplementation. Hence, these may have utility as blood biomarkers of a beneficial epithelial response to supplementation. In addition, the effect on gene expression in blood PBMCs appears robust, since we replicated the effect in a large, independent, expression dataset, namely the BEST-D trial in which subjects were administered oral vitamin D supplementation (2000/4000IU 12 months).

We devised a 2-Phase in vivo approach, firstly to identify differentially expressed genes in the rectal epithelium associated with plasma 25-OHD and determine the GO terms and processes linked to that prioritised gene list. However, our ultimate aim was to establish whether these transcriptomic responses could be recapitulated by oral vitamin D supplementation, thereby demonstrating a modifiable transcriptomic landscape. Many of the top-ranked genes associated with higher 25-OHD level have links with CRC, for instance *CNN1* [36], *COX7A1* [37], PEG3 [62], *PIP5K1C* [38], *TAGLN* [63] and *DAAM2* [64]. Furthermore, we highlight a number of genes within processes relevant to tumourigenesis which are associated with 25-OHD level and influenced by supplementation. The directions of effect of these genes were consistent with tumour suppressor activity. Enrichment of pathways involved in cell migration and cell death validate published in vitro data which demonstrate vitamin D-induced growth arrest and apoptosis of CRC cell lines, modulation of the *Wnt* signalling pathway, DNA repair and immunomodulation [16]. Published clinical data also corroborate our current findings, for example Protiva et al., reported upregulation of genes involved in cell adhesion in response to 1,25(OH)2D3 [17], while Bostick, reported increased cell differentiation and apoptosis in the normal human colorectal epithelium [65]. Taken together, these data suggest possible mechanisms underlying the widely reported link between vitamin D deficiency and increased CRC risk [1, 2]. It also might explain the recently reported beneficial impact of supplementation on CRC survival outcomes [8].

Despite compelling published observational and pre-clinical data, the link between vitamin D and risk of cancer and several other traits remains controversial. Indeed, several large intervention trials have shown no benefit on cancer endpoints (VITAL Trial [66], Vitamin D Assessment (ViDA) study [67, 68] and Baron et al. [69]). However, participants in these trials were predominantly sufficient for vitamin D at the trial outset, thereby potentially blunting beneficial effects [70]. We have previously rehearsed potential reasons why previous study designs might have failed to detect real effects [70]. To counter potential confounding effects, we conducted a Mendelian randomisation study but this also did not demonstrate a beneficial effect of circulating vitamin D on CRC risk. However, available genetic instrumental variables are weak and explain only a small portion of variance of 25OHD levels [71, 72].

In this study, 18% of participants receiving vitamin D supplementation exhibited a response in the colorectal epithelium (the putative target tissue). Unmeasured variables may account for the marked inter-individual variation in response including ethnic or genetic background, dietary, lifestyle, pharmacological (e.g. concurrent medication) or pathological effects (e.g. unknown viral infection during course of supplementation). Variation in the activity of the vitamin D enzymes or carriers (e.g. *CYP24A1*, *GC* and *CYP27B1* undetected in the current dataset) may impact responses in the mucosa relative to 25-OHD change, and we noted differential *CYP2R1* change in the rectum after supplementation between those with/without a response to our candidate gene-set (FC 0.80 vs*.* 1.04, *P* = 0.007). Irrespective of the cause of inter-individual variation in response, if our hypothesis holds that expression changes translate to cancer endpoints, this low response rate would adversely impact on statistical power of trials conducted to date which have tested the effect of vitamin D supplementation on clinical endpoints. Such trials routinely perform subgroup analyses based on change in circulating 25-OHD level, yet the current study reveals poor correlation between plasma level and mucosal gene expression changes, suggesting non-linear responses to vitamin D may introduce further heterogeneity to clinical endpoints. Future work, including GWAS and machine learning approaches will aim to define whether ‘response’ can be determined at baseline is required.

Crucially, we have identified blood biomarkers that reliably identify participants who respond to vitamin D supplementation by inducing gene expression changes in the target tissue. The value of these biomarkers is replicated in a larger independent expression dataset. Further work is required to assess the utility of respective blood protein assays (e.g. ELISA) and reproducibility of these blood biomarkers in identifying mucosal response across a larger cohort. Nevertheless, these exciting and novel findings provide rationale for a trial of vitamin D and CRC prevention using easily assayed blood gene expression signatures as intermediate biomarkers of response.

Whilst the 2-Phase design of an intervention study informed by our correlative dataset has many positive attributes, the study has a number of limitations. First, the low median level of 25-OHD and narrow positively skewed distribution of 25-OHD in the Phase 1 cohort may have masked some true associations between vitamin D level and gene expression. Failure to identify individual gene significance after adjustment for genome-wide multiple testing may also indicate inadequate sample size, physiological autoregulation maintaining constant gene expression despite differences in circulating 25-OHD, differences between plasma and rectal mucosa concentrations of 25-OHD or 1,25-OHD [73] or heterogeneity in cell type in sampled mucosal tissue.

This intervention study is larger than many published studies of gene expression and vitamin D supplementation [17, 74–77], yet may still have limited power to achieve individual gene significance. Phase 2 participants were recruited as a subset of the Phase 1 cohort, which may influence gene-set enrichment test results. However, we did not select those who received supplementation based on 25-OHD level or baseline gene expression, which could have led to overfitting of the data, but instead took an unselected group. If anything, this approach could blunt the observed effect of supplementation, as mucosal response to supplementation may be capped in those with specific 25-OHD or favorable patterns of gene expression at baseline. Despite this, we observed significant enrichment of the candidate gene-set derived in Phase 1 in those receiving supplementation. Unmeasured variation in environmental exposures (e.g. diet or UVB exposure) may have influenced responses, which should be accurately detailed in future studies of vitamin D supplementation. Sampling of rectal mucosa but not colonic mucosa avoided the use of cleansing bowel laxatives which may influence gene expression [78], yet limits the generalisability of our findings to more proximal colonic mucosa. Sampling after 12 weeks of supplementation may not adequately capture early or later gene expression changes yet more frequent or delayed sampling would provide additional practical and ethical challenges. Finally, we recognise responses to vitamin D supplementation and the biomarker utility of *HIPK2* and *PPP1CC* require further mechanistic study (e.g. protein expression) to validate and expand these findings towards a fuller understanding of both biological mechanisms and biomarker potential.

## Conclusions

In conclusion, we report for the first time patterns of gene expression and functional pathways in the normal rectal mucosa that are associated with circulating plasma vitamin D level. Oral vitamin D supplementation induces transcriptomic changes consistent with beneficial anti-tumour effects. Blood leukocyte expression of *HIPK2* and *PPP1CC* predicted well those participants with the greatest expression response following supplementation. Whilst further replication in a separate cohort is desirable, these data provide compelling rationale for a trial of vitamin D and CRC prevention using easily assayed blood gene expression signatures as intermediate biomarkers of response.

## Supplementary Information

**Additional file 1.** Full protocol for SCOVIDS study.

**Additional file 2.** Supplementary methods to be read as an adjunct to the main methods section in the manuscript.

**Additional file 3: Table S1.** Full list of diagnoses in recruited participants. **Table S2.** Top 50 ranked genes in PHASE 1. **Table S3.** Top 50 ranked GO terms enriched in PHASE 1. **Table S4.** Candidate genes positively associated with 25-OHD level in the GO term ‘regulation of cell migration’ and published evidence of tumour suppressor/ biomarker activity. **Table S5.** Candidate genes positively associated with 25-OHD level in the GO term ‘regulation of programmed cell death’ and published evidence of tumour suppressor/ biomarker activity. **Table S6.** Association between relevant genetic variants and 25OHD fold-change after supplementation. **Table S7.** Direction and magnitude of effect in candidate genes with expression change after supplementation. **Table S8.** Top ranked Enriched GO terms after vitamin D supplementation. **Table S9.** Enrichment of candidate GO terms after supplementation in RNAseq data. **Table S10.** Characteristics of participants with rectal mucosa response to supplementation. **Table S11.** AUC values for blood biomarkers of rectal and blood response in the SCOVIDS and BEST-D studies. **Figure S1.** Gene-set enrichment plot for enrichment of candidate genes associated with 25-OHD level in those undergoing vitamin D supplementation. **Figure S2.** GO terms enriched in PHASE 1 and PHASE 2. **Figure S3.** Receiver-operator curve for putative blood biomarkers and rectal mucosal response to supplementation.

## Data Availability

Transcriptomic profiling available at https://www.ncbi.nlm.nih.gov/geo/ GEO ID GSE157982 [79]. Full phenotypic data available from the corresponding author on reasonable request.

## Abbreviations

25-OHD: 25-Hydroxyvitamin D
AUC: Area under curve
BEST-D: Biochemical Efficacy and Safety Trial of Vitamin D trial
CRC: Colorectal cancer
DNA: Deoxyribonucleic acid
FC: Fold-change
GO: Gene ontology
NM: Normal mucosa
PBMC: Peripheral blood mononuclear cells
RCT: Randomised control trial
RNA: Ribonucleic acid
SCOVIDS: Scottish Vitamin D study
SNP: Single-nucleotide polymorphism
VDR: Vitamin D receptor gene
