# Supplementary material for: Oral vitamin D supplementation induces transcriptomic changes in rectal mucosa that are linked to anti-tumour effects
Source: BMC Med. 2021 Aug 3;19:174. doi: 10.1186/s12916-021-02044-y (PMC8330024; doi:10.1186/s12916-021-02044-y)
Supplement: Supplementary file 1 — Additional file 1. Full protocol for SCOVIDS study. [file 12916_2021_2044_MOESM1_ESM.doc]

####
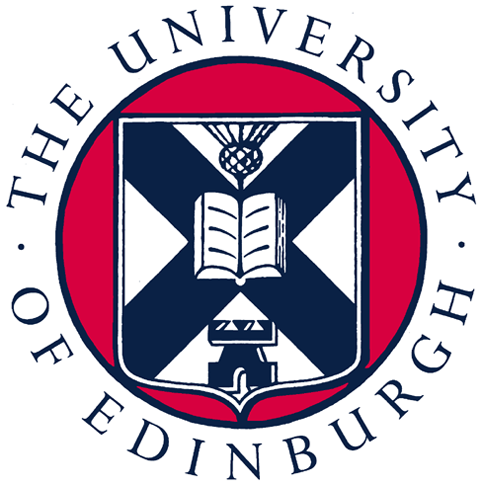

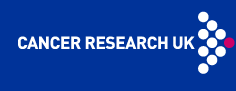

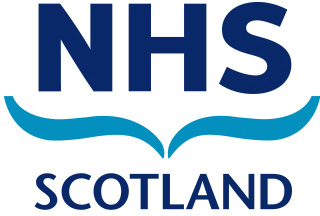


**PROTOCOL**

**Scottish Vitamin D Intervention Study**

**(SCoViDS)**

CO-SPONSORS: The University of Edinburgh and NHS Lothian

STUDY COORDINATING MRC IGMM

CENTRE: University of Edinburgh

Western General Hospital

Edinburgh

EH4 2XU

REC reference:

R&D reference:

Protocol authorised by: Professor Malcolm Dunlop,

Medical Research Council Human Genetics Unit,

Western General Hospital, Edinburgh EH4 2XU

**Study Management Group**

Chief Investigator: Professor Malcolm Dunlop MBChB MD FRCSE

Professor of Coloproctology

University of Edinburgh

MRC Human Genetics Unit at the MRC IGMM

Western General Hospital

Edinburgh

EH4 2XU

Email: [Malcolm.dunlop@hgu.mrc.ac.uk](mailto:Malcolm.dunlop@hgu.mrc.ac.uk)

Telephone: 0131 467 8439

Co-investigator: Dr Susan M Farrington

Senior Scientist

University of Edinburgh

MRC Human Genetics Unit at the MRC IGMM

Western General Hospital

Edinburgh

EH4 2XU

Email: [Susan.Farrington@igmm.ed.ac.uk](mailto:Susan.Farrington@igmm.ed.ac.uk)

Telephone: 0131 467 8422

**Study Coordinating Centre**

For general enquiries, supply of study documentation, and collection of data, please contact:

Study Coordinator: Donna Markie

Address: MRC Human Genetics Unit

Western General Hospital

Edinburgh

EH4 2XU

Telephone: 0131 332 2471 (Ext: 2402)

Email: [dmarkie@staffmail.ed.ac.uk](mailto:dmarkie@staffmail.ed.ac.uk)

**Clinical Queries**

Clinical queries should be directed to Donna Markie, Study Coordinator, who will direct the query to the appropriate person.

**Sponsor Representative**

For further information regarding sponsorship, please contact the Head of Research Governance:

Marise Bucukoglu

University of Edinburgh

The Queen’s Medical Research Institute

47 Little France Crescent

Edinburgh

EH16 4TJ

Email: marise.bucukoglu@ed.ac.uk

Telephone: 0131 242 9262

**Funder**

Organisation: Cancer Research UK

Address: PO Box 123

London

WC2A 3PX

Telephone: 020 7242 0200

Email: julia.mitchell@cancer.org.uk

This protocol describes the Scottish Vitamin D Intervention Study and provides information about procedures for entering participants. The protocol should not be used as a guide for the treatment of other participants; every care was taken in its drafting, but corrections or amendments may be necessary. These will be circulated to investigators in the study, but centre entering participants for the first time are advised to contact the study co-coordinator to confirm they have the most recent version. Problems relating to this study should be referred to the study coordinating centre.

This study will adhere to the principles set out in the International Conference on Harmonisation Good Clinical Pratice (ICH-GCP) guidelines. It will be conducted in compliance with the protocol, the Data Protection Act and Caldicott Standards.

Table of Contents

[1. INTRODUCTION 8](#__RefHeading___Toc64736834)

[1.1 BACKGROUND & RATIONALE 8](#__RefHeading___Toc64736835)

[2. STUDY OBJECTIVES 8](#__RefHeading___Toc64736836)

[3. STUDY DESIGN 9](#__RefHeading___Toc64736837)

[3.1 STUDY OUTCOME MEASURES 9](#__RefHeading___Toc64736838)

[4. PARTICIPANT ENTRY 9](#__RefHeading___Toc64736839)

[4.1 INCLUSION CRITERIA 9](#__RefHeading___Toc64736840)

[4.2 EXCLUSION CRITERIA 9](#__RefHeading___Toc64736841)

[4.3 WITHDRAWAL CRITERIA 10](#__RefHeading___Toc64736842)

[5. STUDY METHODOLOGY 10](#__RefHeading___Toc64736843)

[5.1 IDENTIFICATION OF PARTICIPANTS 10](#__RefHeading___Toc64736844)

[5.2 RECRUITMENT OF PARTICIPANTS 10](#__RefHeading___Toc64736845)

[5.3 INFORMED CONSENT 11](#__RefHeading___Toc64736846)

[5.4 PARTICIPATION IN OTHER TRIALS 11](#__RefHeading___Toc64736847)

[5.5 CONCOMITANT MEDICATION 12](#__RefHeading___Toc64736848)

[5.6 LABORATORY SAMPLES 12](#__RefHeading___Toc64736849)

[5.7 VITAMIN D SUPPLEMENTATION 12](#__RefHeading___Toc64736850)

[5.8 DEMOGRAPHIC DATA 13](#__RefHeading___Toc64736851)

[5.9 QUICK GUIDE RECRUITMENT SCHEDULE 13](#__RefHeading___Toc64736852)

[5.10 ( Figure 1 ) QUICK GUIDE RECRUITMENT SCHEDULE 13](#__RefHeading___Toc64736853)

[5.11 (Figure 2) QUICK GUIDE RECRUITMENT SCHEDULE – 18 WEEK PROTOCOL 14](#__RefHeading___Toc64736854)

[5.12 ( Figure 3 ) QUICK GUIDE RECRUITMENT SCHEDULE 14](#__RefHeading___Toc64736855)

[6. POTENTIAL RISKS AND BENFITS OF PARTICIPATION 15](#__RefHeading___Toc64736856)

[6.1 POTENTIAL RISKS 15](#__RefHeading___Toc64736857)

[6.2 POTENTIAL BENEFITS 16](#__RefHeading___Toc64736858)

[7. STATISTICAL ANALYSIS 16](#__RefHeading___Toc64736859)

[7.1 SAMPLE SIZE 16](#__RefHeading___Toc64736860)

[7.2 METHODS OF ANALYSIS 17](#__RefHeading___Toc64736861)

[8. ADVERSE EVENTS 17](#__RefHeading___Toc64736862)

[8.1 DEFINITIONS 17](#__RefHeading___Toc64736863)

[8.2 IDENTIFYING AEs AND SAEs 18](#__RefHeading___Toc64736864)

[If there is any doubt as to whether a clinical observation is an AE, the event will be recorded. 18](#__RefHeading___Toc64736865)

[8.3 RECORDING AEs AND SAEs 18](#__RefHeading___Toc64736866)

[8.4 ASSESSMENT OF AEs AND SAEs 19](#__RefHeading___Toc64736867)

[Assessment of Seriousness 19](#__RefHeading___Toc64736868)

[Assessment of Causality 19](#__RefHeading___Toc64736869)

[Assessment of Expectedness 19](#__RefHeading___Toc64736870)

[Assessment of Severity 19](#__RefHeading___Toc64736871)

[8.5 REPORTING OF SAEs/SARs/SUSARs 20](#__RefHeading___Toc64736872)

[8.5 REPORTING REQUIREMENTS 20](#__RefHeading___Toc64736873)

[8.6 FOLLOW UP PROCEDURES 20](#__RefHeading___Toc64736874)

[9. REGULATORY ISSUES 20](#__RefHeading___Toc64736875)

[9.1 ETHICS APPROVAL 20](#__RefHeading___Toc64736876)

[9.2 INFORMED CONSENT 21](#__RefHeading___Toc64736877)

[9.3 CONFIDENTIALITY 21](#__RefHeading___Toc64736878)

[9.4 DATA PROTECTION 21](#__RefHeading___Toc64736879)

[9.5 INDEMNITY 22](#__RefHeading___Toc64736880)

[9.6 FUNDING 22](#__RefHeading___Toc64736881)

[9.7 STUDY MONITORING AND AUDIT 22](#__RefHeading___Toc64736882)

[10. STUDY MANAGEMENT 22](#__RefHeading___Toc64736883)

[11 REFERENCES 24](#__RefHeading___Toc64736884)

[SCoViDS DATA COLLECTION PROFORMA 25](#__RefHeading___Toc64736885)

[APPENDIX 2 VITAMIN D DIARY 26](#__RefHeading___Toc64736886)

STUDY SUMMARY

| TITLE | Scottish Colorectal Cancer Vitamin D Intervention Study (SCoViDS) |
| --- | --- |
| DESIGN | Through NHS clinical services in colorectal surgery and oncology, patients will be identified and recruited from surgical wards or surgical/oncology out-patient clinics. A sample of participants with and without a new or previous diagnosis of colorectal cancer will be included for comparison.  Participation will consist of two events in the majority of participants. Firstly a in the surgical ward or clinic lasting no longer than 20 minutes in which the research will be discussed and informed consent gained. A blood sample will be taken prior to the conclusion of recruitment and a rectal biopsy taken using a rigid sigmoidoscopy which may or may not be required as part of their routine clinical assessment. Participants will be asked to take pharmaceutical grade vitamin D tablets for 3 months. After 12 weeks of vitamin D supplementation, a final blood sample and rectal biopsy will be taken.  **If patients would like to contribute but cannot or would prefer not to take vitamin D, or cannot return for future sampling, a single sampling will be offered. This participant would undergo blood sampling and rectal biopsy as above. After this no further events would occur.** |
| AIM | To identify the underlying mechanism by which Vitamin D reduces colorectal cancer risk. |
| OBJECTIVES | To demonstrate the effects of vitamin D supplementation on serum vitamin D levels.  To demonstrate dynamic changes in gene expression in response to vitamin D.  To demonstrate the mechanism underlying the gene-environment interaction of vitamin D, susceptibility genetic variants (risk genes) and colorectal cancer. |
| OUTCOME  MEASURES | *Primary outcome:* Change in expression of genes in bowel tissue in response to vitamin D  *Secondary outcomes:*  Change in expression of genes in bowel tissue in response to vitamin D dependent on participant genotype  Change in serum vitamin D level in response to vitamin D supplementation.  Change in tissue vitamin D level in response to vitamin D supplementation. |
| ELIGIBILITY | Aged 16 years or over.  Resident of the UK. |

# 1. INTRODUCTION

# 1.1 BACKGROUND & RATIONALE

Colorectal cancer (CRC) is common, with >1 million annual incidence worldwide and is responsible for large numbers of deaths and considerable morbidity. Prevention is a particularly appealing strategy to combat CRC, but there is a paucity of well-founded mechanistic research.

Vitamin D deficiency has recently been implicated as a major risk factor in CRC aetiology in geographical, cohort and case-control studies. The evidence is strong for association with CRC risk, and with cancer death. However, causality has yet to be established and adequately powered randomised clinical trials have never been conducted.

No previous study has investigated the way vitamin D affects gene expression in the bowel tissue itself and no study has used vitamin D shown a reduction in cancer. While the evidence for vitamin D reducing bowel cancer risk is extremely strong, the concept of ‘reverse causality’ (i.e. that cancer reduces vitamin D levels) cannot be completely ruled out without an interventional study such as is proposed here.

This study aims to uncover the mechanisms via which vitamin D affects bowel cancer risk. Using gene expression as an endpoint rather than cancer incidence means that far fewer study participants are required while the study period can be much shorter.

If we are successful in our aims we will demonstrate the mechanism by which vitamin D reduces bowel cancer risk and support the role of vitamin D use as a public health intervention. Our study will support future work which will contribute to the development of targeted chemoprevention involving vitamin D or related drugs in those at an increased risk of bowel cancer due to their genetic make-up. Finally our study could act as a template for the investigation of other ways environmental factors can affect cancer risk.

# 2. STUDY OBJECTIVES

To demonstrate a change in expression of genes in bowel tissue in response to vitamin D and dependent on genotype at known colorectal cancer risk loci

To demonstrate that vitamin D increases serum and tissue (rectal) vitamin D levels and relate these changes to a change in gene expression.

**To demonstrate a correlation between vitamin D levels and gene expression using single sample measurements.**

To support the development of vitamin D as a preventative agent for bowel cancer

# 3. STUDY DESIGN

# 3.1 STUDY OUTCOME MEASURES

PRIMARY OUTCOME – the change in expression of genes relevant to colorectal cancer risk in response to vitamin D supplementation.

SECONDARY OUTCOMES - the genotypic associations with changes in gene expression in response to vitamin D supplementation, the change in serum and tissue vitamin D level after vitamin D supplementation.

# 4. PARTICIPANT ENTRY

# 4.1 INCLUSION CRITERIA

All participants will be aged 16 years or over.

Participants must be resident in the United Kingdom.

# 4.2 EXCLUSION CRITERIA

The inability to provide informed consent.

Under the age of 16 years.

A non-UK resident.

Patients who may be at increased risk from rigid sigmoidoscopy:

- Individuals who are taking anti-coagulation medication.
- Individuals with platelet disease or other bleeding issues.
- Individuals with a history of a significant rectal bleed.
- Suspected or known bowel perforation
- Anal stenosis
- Acute peritonitis
- Colonic necrosis
- Toxic megacolon
- Acute severe diverticulitis
- Diverticular abscess
- Recent colonic surgery
- Anal fissure
- Severe coagulopathy
- Anticoagulant therapy
- Severe thrombocytopenia
- Severe neutropenia

**Patients who may be at increased risk from Vitamin D supplementation would not be included in the intervention arm but could still be included in the single sample arm:**

- Kidney disease
- High levels of calcium in the blood
- Atherosclerosis
- Sarcoidosis
- Histoplasmosis
- Over-active parathyroid gland (hyperparathyroidism)
- Lymphoma
- Currently taking thiazide diuretics, digoxin or other cardiac glycosides
  - Known allergy to nuts ( as peanut oil contained within vitamin D preparations)
  - Female subjects of child bearing age who are not taking effective contraception during the period of the trial

Patients in whom vitamin D levels may be unpredictable

- Individuals already established on supplementary Vitamin D.
- Individuals recently returned to the UK from an overseas holiday.
- Individuals who have recently lived abroad.
- Patients on anti-epileptic medication

Presence of any of the above exclusion criteria will be elicited from relevant patient records including clinical case notes and recent laboratory tests.

# 4.3 WITHDRAWAL CRITERIA

Study participants are free to withdraw from the study at any time without giving reason. Should consent be withdrawn, the participant and all identifiable data or tissue collected will be withdrawn.

# 5. STUDY METHODOLOGY

# 5.1 IDENTIFICATION OF PARTICIPANTS

Potential participants will be identified by their healthcare team comprising of clinical nurse specialist or surgeon.

Potential participants who have previously been diagnosed with colorectal cancer will be identified through their attendance at out-patients clinic appointments, again by a member of the healthcare team.

# 5.2 RECRUITMENT OF PARTICIPANTS

Potential participants will be given a study information sheet by a member of the healthcare team either when they are on a surgical ward, or when they attend the surgical or oncology out-patients clinic for review.

They will be given appropriate time to read the information, ask questions and decide if they wish to participate. Should potential participants like more time to consider the study, they will be asked to contact the study coordinator once they have made that decision and an appointment will be made to recruit them.

The person recruiting participants will be either a member of the healthcare team or a research nurse from the dedicated research team.

Recruitment will take place in a private area or room within either the ward or clinic. The study will be discussed with participants and their questions and concerns will be addressed prior to gaining informed consent.

# 5.3 INFORMED CONSENT

Written informed consent will be obtained from all participants.

The person responsible for recruitment will explain the research ensuring that the participants have a full understanding of the purpose and nature of the research and what it involves.

The research team will ensure that anyone recruiting participants to this study will be fully trained in obtaining informed consent.

Approach and recruitment of participants within a period of less than 24 hours may sometimes be necessary. Potential participants will still be given time to consider the research before deciding whether or not they wish to participate.

Individuals who might not adequately understand verbal explanations or written information given in English, or who have special communication needs will be offered the appropriate support through the provision of study materials translated into the appropriate language, an interpreter or interpreter for the deaf.

Consent will be sought to keep limited personal data about participant’s longer term. Due to the genetic nature of the research, there is a small possibility that the research will produce information that may be of clinical importance to the patient or their families in the future. Included in the consent form is a clause which seeks participant’s explicit consent detailing whether they wish to be informed of relevant findings or not.

If participants indicate that they wish to be informed about future research findings which may be relevant to them, we will keep personal data relating to their name, address, general practitioner, next of kin or name of a blood relative.

# 5.4 PARTICIPATION IN OTHER TRIALS

Patients currently on clinical trials of treatment will not be recruited into this study.

# 5.5 CONCOMITANT MEDICATION

Patients currently on aspirin therapy or aspirin containing products will have this documented along with the dose and reason for prescription and any other prescribed or medicinal products being taken at the time of recruitment. Patients on already on vitamin D supplementation, or taking thiazide diuretics, digoxin or other cardiac glycosides, or anti-epileptic medication will be excluded.

# 5.6 LABORATORY SAMPLES

A blood sample will be taken into 2 lithium heparin tubes and 2 EDTA tubes by venepuncture (~30mls). The rectal biopsy will be taken into RNA preservative. If patients are required to undergo further endoscopy for clinical reasons, we will request a study biopsy to be taken during this. All samples will be anonymised through use of an identifying number and will be processed by the Wellcome Trust Clinical Research Facility (WTCRF), Western General Hospital, Edinburgh. The research team will make arrangements for the safe transport of samples to the WTCRF through the use of a pre-paid sealed transport system. Following 12 weeks of Vitamin D supplementation repeat blood (~30mls) and rectal biopsies will be taken.

# 5.7 VITAMIN D SUPPLEMENTATION

**Eligible participants in the intervention arm** will be supplied with pharmaceutical grade vitamin D capsules (total dose of 3200iu/ 80mcg per day). If participants show interest in study involvement but are unable or do not want to take vitamin D supplements, they can be recruited to ill undergo identical assessment to other study participants and provide samples to enable assessment for natural variation in serum vitamin D levels and gene expression.

**5.7.1 Vitamin D Supply**

Fultium-D_3_ 800 IU Capsules will be provided by NHS Lothian Pharmacy

Manufactured by:
Jenson Pharmaceutical Services Ltd.
Carradine House
237 Regents Park Road
London
N3 3LF

**5.7.2 Storage**

The product does not require any special temperature storage conditions.

**5.7.3 Summary of Product Characteristics**

The SmPC, last updated on 29^th^ June 2013 will be referred to: http://www.medicines.org.uk/emc/medicine/25664/spc

# 5.8 DEMOGRAPHIC DATA

Any documented occurrence of bowel polyps will be recorded along with any pre-operative and / or post-operative treatment including any short or long course of radiotherapy, chemo-radiation, combination chemotherapy, mono-chemotherapy or palliative chemotherapy in participants with colorectal cancer.

# 5.9 QUICK GUIDE RECRUITMENT SCHEDULE

Please refer to page 14, Figure 1 – Quick Guide Recruitment Schedule.

# 5.10 ( Figure 1 ) QUICK GUIDE RECRUITMENT SCHEDULE

**Identification of Participant**

**↓**

**Eligibility criteria assessed including renal function and calcium level**

**↓**

**Participant approach**

**↓**

**Participant Information Leaflet**

**↓**

**Written Informed Consent**

**↓**

**Blood sample, 2 x lithium heparin, 2 x EDTA and rectal biopsy**

**↓**

**Vitamin D supplementation for 12 weeks**

**↓**

**Return to specific clinic**

**Blood sample, 4 x lithium heparin and rectal biopsy**

**↓**

**Close**

#

# 5.11 (Figure 2) QUICK GUIDE RECRUITMENT SCHEDULE – 18 WEEK PROTOCOL

**Identification of Patient**

**↓**

**Eligibility criteria assessed including renal function and calcium level**

**↓**

**Patient approach**

**↓**

**Patient Information Leaflet**

**↓**

**Written Informed Consent**

**↓**

**Blood sample, 2 x lithium heparin, 2 x EDTA and rectal biopsy**

**↓**

**6 week interval (no vitamin D supplementation)**

**↓**

**Blood sample, 4 x lithium heparin and rectal biopsy**

**↓**

**Vitamin D supplementation for 12 weeks**

**↓**

**Return to specific clinic (WTCRF)**

**Blood sample, 4 x lithium heparin, and rectal biopsy**

**↓**

**Close**

# 5.12 ( Figure 3 ) QUICK GUIDE RECRUITMENT SCHEDULE

**Single Sample Measurement**

**Identification of Participant**

**↓**

**Eligibility criteria assessed including renal function and calcium level**

**↓**

**Participant approach**

**↓**

**Participant Information Leaflet**

**↓**

**Written Informed Consent**

**↓**

**Blood sample, 2 x lithium heparin, 2 x EDTA and rectal biopsy**

**↓**

**Close**

# 6. POTENTIAL RISKS AND BENFITS OF PARTICIPATION

# 6.1 POTENTIAL RISKS

Study participants may experience mild discomfort or bruising from venepuncture. Where possible, arrangements will be made to have the samples drawn at the same time as other routine samples.

Study participants may experience mild discomfort from rigid sigmoidoscopy. Rectal biopsies are routinely taken during colorectal assessment by rigid sigmoidoscopy and where possible, research samples will be taken at the same time as routine assessment. In such cases, patients recruited into the study will consent to a further additional small biopsy of rectal tissue prior to the procedure. Rigid sigmoidoscopy and rectal biopsy is not associated with significant risk of harm. Major risks include perforation, haemorrhage or pelvic sepsis, with a negligible incidence of each of these.

There is the possibility that a participant/ participant’s family will be identified as potentially having a greater risk of developing colorectal cancer and this could increase anxiety. In this instance, the lead Physician/Surgeon coordinating their care will be contacted and advised that it would be appropriate to refer the family to the local genetics service. Arrangements have been put in place with the four Scottish Regional Genetic Centres to ensure that such referrals are streamlined and that families will be seen in a timely manner.

Vitamin D is currently widely available 'over the counter' as a dietary supplement with its benefits and safety profile being well documented. The European Food Safety Authority has reported a no observed adverse effect level of up to 10000iu/day in healthy young men with minimal sun exposure with an upper limit of 4000iu/day set in order to embrace other ages and genders, including pregnant women. The following medical conditions will be elicited and relevant patients excluded from the study:

- Kidney disease
- High levels of calcium in the blood
- Atherosclerosis
- Sarcoidosis
- Histoplasmosis
- Over-active parathyroid gland (hyperparathyroidism)
- Lymphoma

# 6.2 POTENTIAL BENEFITS

**Benefit from participation in medical research**

Individuals agreeing to participate may find contributing to this exciting study to be a very positive experience.

**Benefit from serum blood sampling**

A review of previous blood results may identify renal disease or high calcium levels that would preclude entry into the study but require further management. The participant and relevant health care professionals will be informed of these results. Blood sampling may also identify significant vitamin D deficiency which would require prolonged vitamin D supplementation following the completion of the study.

**Benefit from DNA analysis**

DNA analysis will be limited to genes of known relevance to colorectal cancer. Should any participant possess alleles known to significantly increase colorectal cancer risk, appropriate counselling and further investigation or surveillance will be arranged.

**Benefit from rigid sigmoidoscopy**

Repeat rectal is unlikely to offer limited benefit. There is a small miss-rate associated with rigid sigmoidoscopy, flexible sigmoidoscopy and colonoscopy. Given this, repeat rectal visualisation may identify previously missed pathology, yet given the relatively small numbers of participants, this is unlikely.

**Benefit from vitamin D supplementation**

Therapeutic benefit from study vitamin D supplementation will be limited given the short period over which vitamin D supplements will be given.

# 7. STATISTICAL ANALYSIS

# 7.1 SAMPLE SIZE

Due to the novel nature of this study and methodology, a convention power calculation was not possible. Using genes which have been shown to be of particular interest to the relationship between vitamin D and bowel cancer, our department has calculated the mean expression of these genes to be ~7.5 units on gene expression (HT-12 microarray) analysis. An analysis of 10 genes would require a significance level adjusted for the effect of multiple testing (hence p<0.005) and with a conventional power level set at 80%. The maximum standard deviation on expression data was 0.42. Assuming 1 standard deviation (i.e. more than twice the observed between subject variation the number of subjects required to detect a change of 4 units (approximately 1.5 fold) would be n=8 and 8 units (approximately 2 fold) would be n=5. Assuming 2 standard deviations (i.e. more than four times the observed between subject variation, the number of subjects required to detect a change of 4 units (approximately 1.5 fold) would be n=57 and 8 units (approximately 2 fold) would be n=18.

*In vitro* pilot work to date has demonstrated a 5-7 fold increase in the expression of one gene of particular interest in response to vitamin D treatment which supports the above calculations.

# 7.2 METHODS OF ANALYSIS

Serum vitamin D measurement will be undertaken by Liquid chromatography–mass spectrometry (Glasgow RI Biochemistry). Bowel tissue (rectal biopsy) measurement of vitamin D will be undertaken using a novel assay system reported in the literature (Immunodiagnostic Systems, Scottsdale, AZ, USA).

Genotyping will be undertaken to identify risk alleles using Illumina OmniExpress Exome GWAS arrays which provides comprehensive cost-efficient genotyping of loci of interest and pre and post active treatment gene expression assessed using Illumina HT-12 array and validated using qRT-PCR. Statistical comparison of pre- and post-treatment gene expression will be undertaken using the Mann-Whitney U test.

# 8. ADVERSE EVENTS

The Investigator is responsible for the detection and documentation of events meeting the criteria and definitions detailed below.

Full details of contraindications and side effects that have been reported following administration of Vitamin D can be found in the relevant Summary of Product Characteristics (SmPC).

Participants will be instructed to contact their Investigator at any time after consenting to join the trial if any symptoms develop. All adverse events (AE) that occur after joining the trial must be reported in detail in the Case Report Form (CRF) or AE form. In the case of an AE, the Investigator should initiate the appropriate treatment according to their medical judgment. Participants with AEs present at the last visit must be followed up until resolution of the event.

# 8.1 DEFINITIONS

An **adverse event** (AE) is any untoward medical occurrence in a study participant which does not necessarily have a causal relationship with the study intervention.

An **adverse reaction** (AR) is any untoward and unintended response to the study intervention.

A **serious adverse event** (SAE), **serious adverse reaction** (SAR). Any AE or AR that at any dose:

- results in death of the study participant;
- is life threatening*;
- requires in-patient hospitalisation^ or prolongation of existing hospitalisation;
- results in persistent or significant disability or incapacity;
- consists of a congenital anomaly or birth defect;
- results in any other significant medical event not meeting the criteria above.

*Life-threatening in the definition of an SAE or SAR refers to an event where the participant was at risk of death at the time of the event. It does not refer to an event which hypothetically might have caused death if it were more severe.

^Any hospitalisation that was planned prior to randomisation or clearly relating to a pre-existing condition will not meet SAE criteria. Any hospitalisation that is planned post randomisation will meet the SAE criteria.

# 8.2 IDENTIFYING AEs AND SAEs

All AEs and SAEs will be recorded from the time a participant signs the consent form to take part in the study until the end of the study.

Participants will be asked about the occurrence of AEs/SAEs at every visit during the study. Open-ended and non-leading verbal questioning of the participant will be used to enquire about AE/SAE occurrence. Participants will also be asked if they have been admitted to hospital, had any accidents, used any new medicines or changed concomitant medication regimens.

AEs and SAEs may also be identified via information from support departments e.g. laboratories*.*

Events clearly related to a known underlying medical condition, in particular colorectal cancer, will not be recorded, including but not limited to admissions for planned or emergency surgery, admissions with bowel obstruction or admissions with rectal bleeding.

# If there is any doubt as to whether a clinical observation is an AE, the event will be recorded.

# 8.3 RECORDING AEs AND SAEs

When an AE/SAE occurs, it is the responsibility of the Investigator to review all documentation (e.g. hospital notes, laboratory and diagnostic reports) related to the event. The Investigator will then record all relevant information in the CRF and on the SAE form (if the AE meets the criteria of serious).

Information to be collected includes dose, type of event, onset date, Investigator assessment of severity and causality, date of resolution as well as treatment required, investigations needed and outcome.

# 8.4 ASSESSMENT OF AEs AND SAEs

Seriousness, causality, severity and expectedness will be assessed by the Principal Investigator.

The Investigator is responsible for assessing each AE.

The Chief Investigator (CI) may not downgrade an event that has been assessed by an Investigator as an SAE, but can upgrade an AE to an SAE or SAR if appropriate.

### Assessment of Seriousness

The Investigator will make an assessment of seriousness as defined in Section 8.1.

### Assessment of Causality

The Investigator will make an assessment of whether the AE/SAE is likely to be related to the study intervention according to the definitions below.

- Unrelated: where an event is not considered to be related to the study intervention.
- Possibly Related: The nature of the event, the underlying medical condition, concomitant medication or temporal relationship make it possible that the AE has a causal relationship to the study intervention. The assessment of causality will be made against the reference safety information found in the Summary of Product Characteristics [last updated 29^th^ June 2013].

### Assessment of Expectedness

If an event is judged to be related to the study intervention, the evaluation of expectedness will be made based on knowledge of the reaction and the relevant product information documented in the SmPC.

The event may be classed as either:

**Expected**: the AR is consistent with the toxicity of the product listed in the SmPC.

**Unexpected**: the AR is not consistent with the toxicity in the SmPC.

### Assessment of Severity

The Investigator will make an assessment of severity for each AE/SAE and record this on the CRF or SAE form according to one of the following categories:

**Mild**: an event that is easily tolerated by the participant, causing minimal discomfort and not interfering with every day activities.

**Moderate**: an event that is sufficiently discomforting to interfere with normal everyday activities.

**Severe**: an event that prevents normal everyday activities.

Note: the term ‘severe’, used to describe the intensity, should not be confused with ‘serious’ which is a regulatory definition based on participant/event outcome or action criteria. For example, a headache may be severe but not serious, while a minor stroke is serious but may not be severe.

# 8.5 REPORTING OF SAEs/SARs/SUSARs

Once the Investigator becomes aware that an SAE has occurred in a study participant, the information will be reported to the ACCORD Research Governance & QA Office **immediately or within 24 hours**. If the Investigator does not have all information regarding an SAE, they should not wait for this additional information before notifying ACCORD. The SAE report form can be updated when the additional information is received.

The SAE report will provide an assessment of causality and expectedness at the time of the initial report to ACCORD according to Sections titled, Assessment of Causality and Assessment of Expectedness.

The SAE form will be transmitted by fax to ACCORD on **+44 (0)131 242 9447** or may be transmitted by hand to the office or submitted via email to [Safety.Accord@ed.ac.uk](mailto:Safety.Accord@ed.ac.uk). Only forms in a pdf format will be accepted by ACCORD via email.

Where missing information has not been sent to ACCORD after an initial report, ACCORD will contact the investigator and request the missing information.

All reports faxed to ACCORD and any follow up information will be retained by the Investigator in the Investigator Site File (ISF).

# 8.5 REPORTING REQUIREMENTS

The ACCORD Research Governance & QA Office is responsible for reporting SAEs that are considered to be ‘possibly related’ and ‘unexpected’ to the REC on behalf of the co-sponsors (Edinburgh University and NHS Lothian).

An Annual Safety Report will be submitted, by ACCORD to the REC listing all SARs.

# 8.6 FOLLOW UP PROCEDURES

After initially recording an AE or recording and reporting an SAE, the Investigator will follow each participant until resolution or death of the participant. Follow up information on an SAE will be reported to the ACCORD office.

AEs still present in participants at the last study visit will be monitored until resolution of the event or until no longer medically indicated.

# 9. REGULATORY ISSUES

# 9.1 ETHICS APPROVAL

The Chief Investigator will obtain approval for this research from the South East Scotland Research Ethics Committee via the IRAS system.

**9.2 INVESTIGATOR RESPONSIBILITIES**

The Investigator is responsible for the overall conduct of the study at the site and compliance with the protocol and any protocol amendments.

# 9.2 INFORMED CONSENT

The Investigator is responsible for ensuring informed consent is obtained before any protocol specific procedures are carried out. The decision of a participant to participate in clinical research is voluntary and should be based on a clear understanding of what is involved.

Participants must receive adequate oral and written information – appropriate Participant Information and Informed Consent Forms will be provided. The oral explanation to the participant will be performed by the Investigator or qualified delegated person, and must cover all the elements specified in the Participant Information Sheet and Consent Form.

The participant must be given every opportunity to clarify any points they do not understand and, if necessary, ask for more information. The participant must be given sufficient time to consider the information provided. It should be emphasised that the participant may withdraw their consent to participate at any time without loss of benefits to which they otherwise would be entitled.

The participant will be informed and agree to their medical records being inspected by representatives of the sponsor(s) but understand that their name will not be disclosed outside the hospital.

The Investigator or delegated member of the trial team and the participant will sign and date the Informed Consent Form(s) to confirm that consent has been obtained. The participant will receive a copy of this document and a copy filed in the Investigator Site File (ISF) and participant’s medical notes.

# 9.3 CONFIDENTIALITY

All laboratory specimens, evaluation forms, reports, and other records must be identified in a manner designed to maintain participant confidentiality. All records must be kept in a secure storage area with limited access. Clinical information will not be released without the written permission of the participant. The Investigator and study site staff involved with this study may not disclose or use for any purpose other than performance of the study, any data, record, or other unpublished, confidential information disclosed to those individuals for the purpose of the study. Prior written agreement from the sponsor or its designee must be obtained for the disclosure of any said confidential information to other parties.

# 9.4 DATA PROTECTION

All Investigators and study site staff involved with this study must comply with the requirements of the Data Protection Act 1998 with regard to the collection, storage, processing and disclosure of personal information and will uphold the Act’s core principles. Access to collated participant data will be restricted to those clinicians treating the participants, representatives of the sponsor(s) and representatives of regulatory authorities.

Computers used to collate the data will have limited access measures via user names and passwords.

Published results will not contain any personal data that could allow identification of individual participants.

# 9.5 INDEMNITY

The co-sponsors are responsible for ensuring proper provision has been made for insurance or indemnity to cover their liability and the liability of the Chief Investigator and staff.

The following arrangements are in place to fulfil the co-sponsors' responsibilities:

- The Protocol has been designed by the Chief Investigator and researchers employed by the University and collaborators. The University has insurance in place (which includes no-fault compensation) for negligent harm caused by poor protocol design by the Chief Investigator and researchers employed by the University.
- Sites participating in the study will be liable for clinical negligence and other negligent harm to individuals taking part in the study and covered by the duty of care owed to them by the sites concerned. The co-sponsors require individual sites participating in the study to arrange for their own insurance or indemnity in respect of these liabilities.
- Sites which are part of the United Kingdom's Nation Health Service will have the benefit of NHS Indemnity.
- Sites out with the United Kingdom will be responsible for arranging their own indemnity or insurance for their participation in the study, as well as for compliance with local law applicable to their participation in the study.

# 9.6 FUNDING

Cancer Research UK, PO Box 123, London WC2A 3PX. Telephone: 0203469 6400, Email: [Julia.mitchell@cancer.org.uk](mailto:Julia.mitchell@cancer.org.uk).

# 9.7 STUDY MONITORING AND AUDIT

An ACCORD Clinical Trials Monitor or an appointed monitor may visit the Investigator site prior to the start of the study and during the course of the study if required, in accordance with the monitoring plan if required. Risk assessment will determine if audit, by the ACCORD QA group, is required. Details will be captured in an audit plan. Audit of Investigator sites, study management activities and study collaborative units, facilities and 3rd parties may be performed.

# 10. STUDY MANAGEMENT

The management of this study will be coordinated by Donna Markie, Study Coordinator, MRC Human Genetics Unit, Western General Hospital, Edinburgh EH4 2XU. Telephone: 0131 332 2471 (Ext: 2402)

Email: [dmarkie@staffmail.ed.ac.uk](mailto:dmarkie@staffmail.ed.ac.uk)

# SCoViDS DATA COLLECTION PROFORMA

For completion by researcher

**Name**: ……………………………….…..……………

MD:

**Address**: ………………………….….….……………

**Male / Female** …………………………………………………………

**Ethnicity:**…………………. **DOB**: ………………..………

**Participant involved in any previous genetic studies?** Yes / No / Unsure

**Takes regular aspirin? 🞎** Yes **🞎** No

(or product containing aspirin)

If yes, reason …………. Dose: .mg …………….

**Previous or new diagnosis of colorectal cancer? 🞎** Yes **🞎** No

Date (year) ………………

**Consultant**: …………………………………………

**GP details**: ..…………………………………………………………………………………………….

**New or previous diagnosis of colorectal cancer?** Yes / No

**Date of surgery**: ……..………………… **or** / no surgery planned.

**Is this a 2^nd^ primary colorectal tumour?**  Yes / No

**Is this a recurrence?**  Yes / No

**Pre-op treatment**: No / Short course XRT */ Long course XRT **/ Long course chemoradiation

**Post-op treatment** : No / combination chemo / mono chemo / palliative chemo / radiotherapy/ undetermined

***Definitions:*** ** Short course XRT occurs in the 5 days leading up to the day operation ** Long course is 6 weeks +/- chemotherapy, occurring in the 3-4 months prior to operation*

# APPENDIX 2 VITAMIN D DIARY

Please tick or cross when you have taken your vitamin D tablet for the day.

It is best to take at the same time each day, but if you forget it is ok to take later that day.

If you forget one day’s dose, leave the box blank and **do not take two doses the next day**.

| Week | Date | Monday | Tuesday | Wednesday | Thursday | Friday | Saturday | Sunday |
| --- | --- | --- | --- | --- | --- | --- | --- | --- |
| e.g. | 6/1/14 | x | x |  | x | x | x | x |
| 1 |  |  |  |  |  |  |  |  |
| 2 |  |  |  |  |  |  |  |  |
| 3 |  |  |  |  |  |  |  |  |
| 4 |  |  |  |  |  |  |  |  |
| 5 |  |  |  |  |  |  |  |  |
| 6 |  |  |  |  |  |  |  |  |
| 7 |  |  |  |  |  |  |  |  |
| 8 |  |  |  |  |  |  |  |  |
| 9 |  |  |  |  |  |  |  |  |
| 10 |  |  |  |  |  |  |  |  |
| 11 |  |  |  |  |  |  |  |  |
| 12 |  |  |  |  |  |  |  |  |
| 13 |  |  |  |  |  |  |  |  |
| 14 |  |  |  |  |  |  |  |  |
| 15 |  |  |  |  |  |  |  |  |
| 16 |  |  |  |  |  |  |  |  |
| 17 |  |  |  |  |  |  |  |  |
| 18 |  |  |  |  |  |  |  |  |
| 19 |  |  |  |  |  |  |  |  |
| 20 |  |  |  |  |  |  |  |  |
| 21 |  |  |  |  |  |  |  |  |
| 22 |  |  |  |  |  |  |  |  |
| 23 |  |  |  |  |  |  |  |  |
| 24 |  |  |  |  |  |  |  |  |
| 25 |  |  |  |  |  |  |  |  |
| 26 |  |  |  |  |  |  |  |  |
